# Integration of Multi‐Cereals Into Wheat Flour: Nutritional Composition, Dough Rheology, and Sensory Profile of Composite Breads

**DOI:** 10.1002/fsn3.71267

**Published:** 2025-11-26

**Authors:** Farooq Hafeez, Muhammad Tauseef Sultan, Hassan Raza, Muhammad Usman Khalid, Ali Ikram, Muhammad Tayyab Arshad, Iqra Baig, Sidra Imtiaz, Muhammad Abrar, Ali Imran, Kodjo Théodore Gnedeka

**Affiliations:** ^1^ College of Biosystems Engineering and Food Science, Zhejiang Key Laboratory of Agro‐Food Resources and High‐Value Utilization Zhejiang University Hangzhou China; ^2^ Faculty of Food Science and Nutrition Bahauddin Zakariya University Multan Pakistan; ^3^ University Institute of Food Science and Technology The University of Lahore Lahore Pakistan; ^4^ Functional Food and Nutrition Program, Center of Excellence in Functional Foods and Gastronomy, Faculty of Agro‐Industry, Prince of Songkla University Hatyai, Songkhla Thailand; ^5^ Ayub Agriculture Research Institute Faisalabad Pakistan; ^6^ Department of Food Sciences, Government College University Faisalabad Faisalabad Pakistan; ^7^ Togo Laboratory: Applied Agricultural Economics Research Team (ERE2A) University of Lomé Lome Togo

**Keywords:** composite flour, dough rheology, minor cereals, multi‐grain bread, nutritional fortification, nutritional value

## Abstract

Bread, one of the most widely consumed staple foods, is traditionally prepared from whole wheat flour owing to its high gluten content and desirable physiochemical properties. However, growing consumer demand for nutrient‐dense functional foods has prompted researchers to explore composite flour formulations by integrating minor cereals such as barley, oat, corn, millet and sorghum. The present study was aimed at investigating the nutritional composition, dough rheology and sensory attributes of composite bread prepared using different blending ratios. Nine experimental blends were developed including a control group containing whole wheat (100%). Results showed that incorporation of the FB‐4 flour blend containing oats showed improved crude protein (12.61% ± 0.255%), fiber (3.19% ± 0.08%) and ash (0.45% ± 0.009%). Similarly, FB‐5 showed the highest calcium, zinc and iron values (926.50 ± 8.71 ppm; 1.05 ± 0.06 ppm; 1.86 ± 0.70 ppm) respectively. However, the FB‐3 blend with millet exhibited greater heavy metal (0.60 ± 0.40 ppm; 0.97 ± 0.85 ppm) for lead and nickel respectively. FB‐1 (barley blend) showed the highest loaf volume (8.90 ± 0.30) and desirable crust color (7.30 ± 0.31) reflecting improved texture and flavor. Sensory evaluation using a 9‐point hedonic scale showed overall acceptable scores particularly for FB‐4 (oat blend) and FB‐1 (barley blend). The study concludes that partial substitution of flour blends up to 25% can yield health‐oriented baked products, promoting the utilization of minor cereals with high climate resilience.

## Graphical abstract

The current study aimed to explore the integration of selected multi‐cereals, that is, barley, oats, corn, millet and sorghum into wheat flour to produce composite breads. The primary objective is to evaluate nutritional profiling, dough rheological analyses and sensory attributes of developed products to assess consumer acceptance. The research focuses on bridging the gap between traditional cereal use and diverse and functional use in the baking industry.

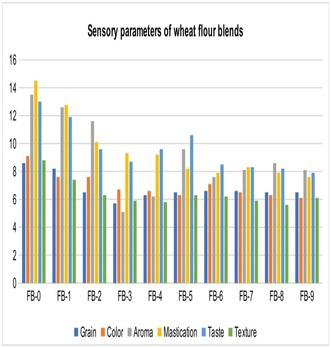

## 1 Introduction

Composite flour consumption in bread making is gaining attention globally due to its nutritional benefits and functional advantage. Cereals are considered a staple diet globally, primarily for their high energy density which is important for long‐term health outcomes. Literature suggests that cereals contribute substantially to the recommended dietary allowance (RDA) for protein in many diets (Safdar et al. 2023). The global cereal production for the 2023–2024 period recorded an output of 2.836 billion tons, including 788 million tons of wheat, 833 million tons of maize, 723 million tons of rice (FAOSTAT 2023). Wheat and rice are considered indispensable cereals forming the backbone of the baking industry. Wheat, in particular, remains a major dietary staple that provides significant carbohydrates, proteins, vitamins and minerals (Tufail et al. 2023). In addition to these staples, minor grains such as wheat, corn, rice barley, and millet are also cultivated in various agro‐climatic zones with high tolerance to climate stressors (Wang et al. 2023). Corn accounts for approximately 31% of global cereal production, while wheat and rice collectively account for 55% of the total cereal output (Li et al. 2017). However, these minor cereals are underutilized due to a lack of gluten matrix which undermines the rheological behavior of dough. Over‐dependence on staples gradually led to micronutrient deficiencies and a rising prevalence of non‐communicable diseases, Moreover, drastic changes in climate threaten the availability of major cereal crops to consumers which urged researchers to explore the potential of underutilized indigenous cereal species (Lafiandra et al. 2014).

Bread is consumed globally due to its easy availability, high nutritional value and affordability. Wheat is considered a dominant crop in the baking industry due to its high gluten content, ideal for bread‐making applications. Wheat‐based bakery products have been used for 12,000 years across different cultures, especially Egypt (Li et al. 2017). With modern advancements in food technology, composite flour formulation has exerted a positive impact on consumer health and reduced dependency on wheat flour only. Moreover, the substitution of wheat flour with minor cereals provides a therapeutic approach for gluten‐intolerant persons. Bread formulations with the addition of enzymes, emulsifiers, colors, and flavors, further encourage the integration of other grains (Qi et al. 2025), that is, barley, millet, oats, sorghum into wheat‐based bakery products (Fuentes and Serna‐Saldivar 2022; Wang and Jian 2022). Minor cereals such as barley, oats, corn, millet and sorghum offer a rich nutritional profile with dietary fiber, minerals and phytochemicals (Imam 2021). Consumer acceptance of depends on their flavor, texture and appearance (Guan et al. 2025). Thus, it is essential to carry out comprehensive evaluations of the nutritional, functional, and sensory attributes (Adeyeye et al. 2019; Li et al. 2025).

The current study aimed to explore the integration of selected multi‐cereals, that is, barley, oats, corn, millet and sorghum into wheat flour to produce composite breads. The primary objective is to evaluate the nutritional profiling, dough rheological analyses and sensory attributes of developed products to assess consumer acceptance. The research focuses on bridging the gap between traditional cereal use and diverse and functional use in the baking industry.

## 2 Materials and Methodology

The present study was conducted in the Department of Human Nutrition, Faculty of Food Science and Nutrition, BZU, Multan, Pakistan. The primary objective was to evaluate the nutritional and rheological properties of fortified bread prepared by incorporating selected cereal powder into wheat flour.

### 2.1 Procurement of Raw Material

Locally available cereals, that is, barley, corn, millet, oat, sorghum were procured from local grocery stores of Multan, Pakistan. All the chemicals and reagents used in the study were of analytical grade and they were sourced from Sigma Aldrich (United Kingdom).

### 2.2 Preparation of Cereal Powder

All raw cereals were cleaned manually and dried in oven at 70°C for 12 h. The dried materials were then milled, sieved and packed in plastic zipper bags for further analysis.

### 2.3 Preparation of Wheat Flour Blends Supplemented With Multi‐Cereals

Nine blends were prepared by mixing different cereals powder in wheat flour. Flour was prepared by homogenous concentration of different cereals (25%) in wheat flour (75%). Treatments of multi‐cereal dough is shown in Table 1.

**TABLE 1:** Treatments of multi‐cereal dough (per 100 g).

FB‐0	100% SGF (single grain flour)
FB‐1	75% SGF + 25% Barley
FB‐2	75% SGF + 25% corn
FB‐3	75% SGF + 25% Millet
FB‐4	75% SGF + 25% Oat
FB‐5	75% SGF + 25% Sorghum
FB‐6	75% SGF + 25% Other (70% Barley + 10% Corn + 10% Millet + 10% Oat)
FB‐7	75% SGF + 25% Other (70% Barley + 10% Corn + 10% Millet + 10% Sorghum)
FB‐8	75% SGF + 25% Other (70% Barley + 10% Millet + 10% Oat + 10% Sorghum)
FB‐9	75% SGF + 25% Other (20% Barley + 20% Corn + 20% Millet + 20% Oat + 20% Sorghum)

### 2.4 Proximate Analysis of Raw Material

The proximate analysis of each blend of wheat flour supplemented with different cereal powders, that is, barley, corn, millet, oat, and sorghum, was determined by AACC (2000) International Approved Methods. Moisture was determined by using a thermostatically controlled hot air oven BST/HAO‐1134 at 105°C. Ash contents were analyzed using a muffle furnace SHF‐FU‐7 for 4 h at 550°C to obtain a gray residue. Crude fat contents were checked by using Soxhlet apparatus (402288, Moravia, Czech Republic). For fiber, samples were initially treated with dilute H_2_SO_4_ (1.25%), rinsed to wash out acid residues and then with dilute KOH solution (1.25%), allowed to boil for 30 min. After cooling, residues were placed in a muffle furnace for 4 h at 550°C to obtain white ash. Protein contents were determined by using Kjeldahl distillation apparatus (ELE‐216EP3, Copenhagen, Denmark). Carbohydrates were calculated by subtracting the sum of moisture, fat, fiber, protein and ash from 100. Results were expressed in percentages.

### 2.5 Mineral Composition

Mineral composition including Calcium, Potassium, Sodium, Zinc, Iron, Lead and Nickel contents of different cereals was evaluated by following the procedure of Carocho et al. (2020). Dry ash mineralization of samples was done at 550°C. Expression of the result is in ppm of the fresh weight.

### 2.6 Dough Rheological Properties

The dough rheological properties of different wheat flour samples were measured by running through Brabander Farinograph and Brabander Extensograph (Brabender D‐4100 SEW; Germany) according to their respective methods described in AACC (2000) method No. 54‐21. The farinograms were illuminating for different characteristics like water absorption, dough development time, dough stability, mixing tolerance index and softening of the dough. Extensograms were also carried out according to (Arshad et al. 2025).

### 2.7 Preparation of Multi‐Cereal Bread

For bread making, the standard procedure outlined by Saddique et al. (2024) was followed. Straight grade wheat flour was partially replaced by selected cereals, that is, barley, corn, millet oat and sorghum in various combinations as shown in Table 1. After thorough mixing, dough molding was carried out and then placed in a proofer having a relative humidity of 80% at 35°C for 45 min. The loaves were baked for 30–35 min at a temperature of 220°C–230°C. After baking, the loaves were cooled to room temperature and packed in food grade polythene bags to maintain moisture. The composite bread was stored under ambient storage conditions (25%) until further analysis. Regular monitoring for changes in color, odor and firmness was carried out to assess shelf‐life and acceptability.

### 2.8 Physical Characteristics

Bread prepared by using multi‐cereals was analyzed for determination of bread loaf volume (cm^3^), bread density (kg/m^3^) as described in AACC (2000), bread symmetry, and crust. Color analysis of bread crust and crumb was also observed by using a calorimeter (Konic Minolta, CR‐5, Japan) which measured the values in terms of lightness, yellowness, bluishness, chroma and hue of the different treatments of multi‐cereal bread dough.

### 2.9 Texture Profile

To determine the texture profile of bread, the Plus Texture Analyzer was used to determine texture parameters namely adhesiveness, hardness, cohesiveness and springiness with a test speed of 3 mm/s and 25% strain on it.

### 2.10 Sensory Evaluation

Multi‐cereal breads were let to cool and sliced 1–2 h after baking after placing them at room temperature. Bread slices were set to put in disposable plates with random numbers assigned. Bread samples were evaluated for descriptive sensory/organoleptic evaluation by 25 trained panelists with the best treatment scoring 9 and the least desirable treatment scoring 1 on a nine‐point hedonic scale. Consent Form for the consumption was duly filled out by panelists. All the trained panelists were given an ambient environment and they were provided water for rinsing between the samples before the next sample. The parameters that were evaluated in the sensory evaluation were aroma, crust color, flavor, texture, mouthfeel, and chew ability (Larmond 1977).

### 2.11 Statistical Analysis

All experiments were performed in triplicates to attain accuracy. The data were statistically analyzed using CRD design on Statistix 81. The results were represented as mean and standard deviations. ANOVA testing was used to test the significant differences. Post Tukey test was used to compare the means of different parameters.

## 3 Results and Discussion

### 3.1 Proximate Analysis of Raw Materials

Proximate Analysis of selected cereals presented in (Table 2), showed the nutritional profile of each grain, highlighting their diverse role in the food industry. Among the selected grains, wheat and oat indicated comparatively higher moisture content (14.30% ± 0.34%, 11.09% ± 0.31%, 7.84% ± 0.51%), respectively. In contrast, barley, corn, and sorghum showed comparatively lower moisture levels, while, sorghum recorded minimum moisture (6.35% ± 0.42%), depicting more suitability for prolonged storage. Highest ash contents were noted in oats (0.89% ± 0.03%), followed by sorghum (0.86% ± 0.02%) and corn (0.29% ± 0.02%), highlighting their rich mineral contents. Fat contents were presented in a similar trend, with oats containing the highest fat contents (6.23% ± 0.17%) followed by sorghum (2.48% ± 0.14%) and barley (2.16% ± 0.05%). In contrast, corn and wheat showed the lowest fat content (1.51% ± 0.09 and 1.32% ± 0.02%) respectively. In terms of crude fiber, oats showed the highest fiber content (12.77% ± 0.34%), followed by wheat (0.251% ± 0.011%) and sorghum (6.12% ± 0.36%). However, millet recorded the lowest crude fiber (2.78% ± 0.17%). Nitrogen Free Extract (NFE), representing digestible carbohydrates showed maximum values in barley (76.72% ± 1.58). Overall trend indicates that oats are rich in ash, fiber and fat contents, while barley and wheat exhibited higher carbohydrates. Sorghum however, presented low moisture levels and moderate carbohydrates.

**TABLE 2:** Proximate composition (%) of multi‐cereals.

Proximate %	Wheat	Barley	Corn	Millet	Oat	Sorghum	IPM
Moisture	14.30 ± 0.34	7.65 ± 0.16	7.42 ± 0.49	7.84 ± 0.51	11.09 ± 0.31	6.35 ± 0.42	7.17 ± 0.12
Ash	0.32 ± 0.006	0.23 ± 0.005	0.29 ± 0.02	0.38 ± 0.02	0.89 ± 0.03	0.86 ± 0.02	0.38 ± 0.01
Fat	1.32 ± 0.02	2.16 ± 0.05	1.51 ± 0.09	1.52 ± 0.09	6.23 ± 0.17	2.48 ± 0.14	1.85 ± 0.03
Protein	12.40 ± 0.27	11.27 ± 0.23	7.05 ± 0.46	10.5 ± 0.59	13.23 ± 0.34	7.29 ± 0.46	22.26 ± 0.44
Fiber	0.251 ± 0.011	7.74 ± 0.15	3.19 ± 0.21	2.78 ± 0.17	12.77 ± 0.34	6.12 ± 0.36	1.76 ± 0.03
NFE	71.73 ± 1.62	76.72 ± 1.58	74.56 ± 4.92	52.04 ± 3.12	53.16 ± 1.32	73.34 ± 4.64	52.02 ± 3.10

Abbreviation: IPM, Industrial pre mix.

Findings of Alemayehu et al. (2023), validated that the rich dietary fiber composition of wheat flour supplemented with oat provides a rich amount of β‐glucan, while the lack of gluten provides improved elasticity. Moreover, the high fat and fiber content in wheat flour supplemented with oat, which in turn, improved digestibility, energy score and enhanced absorption.

### 3.2 Minerals Analysis in Different Cereals

Table 3, presents significant variations in macro and micro mineral concentrations among the selected grains, which highlighted their nutritional differences. Highest calcium contents were found in wheat and sorghum (977.66 ± 5.65 and 773.32 ± 2.86 ppm), respectively, while, millet contained the lowest calcium contents (516.66 ± 7.37 ppm). Similarly, notable differences were seen in potassium levels, with wheat carrying highest contents (848.33 ± 4.00 ppm), followed by millet and sorghum, whereas, oats showed minimum potassium contents (507.33 ± 5.37 ppm). Likewise, millet showed greatest sodium contents (973.33 ± 6.31 ppm), followed by wheat, while sorghum showed minimum sodium (546.33 ± 5.27 ppm), compared to other grains. For trace minerals, sorghum had the greatest zinc concentration (2.57 ± 0.16 ppm), followed by millet and barley, whereas, wheat showed least zinc levels (0.53 ± 0.03 ppm). Following a similar trend, highest iron levels were noted in corn (5.24 ± 0.32 ppm), followed by oats and sorghum. However, wheat again showed lowest iron levels (2.20 ± 0.13 ppm). The concentration of heavy metals was noted to have significant variations in selected grains. Lead showed highest concentration in oats (0.16 ± 0.010) and lowest levels in sorghum (0.04 ± 0.003 ppm). Similarly, wheat showed highest nickel levels (0.20 ± 0.010 ppm) and lower levels were found in oats (0.005 ± 0.00 ppm), ranging within the acceptable limits. Overall, mineral composition (ppm) of selected grains is presented in Table 3. The findings were endorsed by Oluseyi et al. (2021) who reported richer iron and calcium contents in corn. These results are in accordance with Raya et al. (2022) analyzed higher zinc content in corn flour (1.68 mg/100 g) than that in wheat flour (72% ext.) (0.66 mg/100 g).

**TABLE 3:** Mineral composition (ppm) of wheat flour blends supplemented with multi‐cereals.

Cereals	Calcium (ppm)	Potassium (ppm)	Sodium (ppm)	Zinc (ppm)	Iron (ppm)	Lead (ppm)	Nickel (ppm)
Wheat	977.66 ± 5.65	848.33 ± 4.00	875.33 ± 4.11	0.53 ± 0.03	2.20 ± 0.13	0.14 ± 0.009	0.20 ± 0.010
Barley	636.66 ± 3.85	777.00 ± 6.76	798.33 ± 7.52	1.50 ± 0.09	2.41 ± 0.15	0.09 ± 0.005	0.01 ± 0.000
Corn	548.66 ± 4.13	656.00 ± 9.08	695.33 ± 7.09	1.29 ± 0.00	5.24 ± 0.32	0.13 ± 0.008	0.01 ± 0.001
Millet	516.66 ± 7.37	760.33 ± 6.35	973.33 ± 6.31	2.01 ± 0.12	2.62 ± 0.15	0.10 ± 0.006	0.01 ± 0.001
Oat	651.66 ± 5.51	507.33 ± 5.37	874.54 ± 4.63	1.24 ± 0.07	1.85 ± 0.11	0.16 ± 0.010	0.005 ± 0.00
Sorghum	773.32 ± 2.86	657.94 ± 5.70	546.33 ± 5.27	2.57 ± 0.16	2.57 ± 0.16	0.04 ± 0.003	0.004 ± 0.00

### 3.3 Proximate Content of Wheat Flour Blends (%)

The proximate analysis of wheat flour blends supplemented with selected cereals presented in (Table 4) showed noticeable variations in their nutritional composition, when compared with standard wheat flour. Moisture level ranged from 12.31% to 14.31%, with comparatively higher values in FB‐0, FB‐3, and FB‐4 blends (14.31% ± 0.322%, 12.69% ± 0.333%, 13.52% ± 0.278%) respectively. Ash contents varied from 0.29% to 0.45% with slightly elevated ash contents in FB‐4 (0.45% ± 0.009%) blend. Fat levels ranged from about 1.30% to 2.53% with the highest fat content recorded in FB‐4 blend (2.53% ± 0.053%). Fluctuations in protein contents ranged from 11.07% to 12.61% without following a consistent pattern. Meanwhile, the crude fiber showed a wide range between 0.25% and 3.19%. On a similar trend, blend FB‐4 showed a maximum crude fiber value (3.19% ± 0.082%). Likewise, Nitrogen Free Extract (NFE) ranged from about 66.80% to 72.96% indicating slight variations in digestible carbohydrate proportion among different treatment blends. Wheat flour supplemented with buckwheat flour showed higher fiber content with reduced starch which in turn, increases satiety and lowers post prandial glucose (Kowalski et al. 2022). A study conducted by Chauhan et al. (2018) incorporated 20% oat flour with wheat flour for bread preparation which enhanced the overall nutritional profile of bread. Similarly, carbohydrates and phytate content were reduced significantly in soaked barley flour which improves the digestibility of flour dough (Abdullah et al. 2025).

**TABLE 4:** Proximate composition (%) wheat flour blends supplemented with multi‐cereal bread.

Flour blend	Moisture %	Ash %	Fat %	Protein %	Fiber %	NFE %
FB‐0	14.31 ± 0.322^a^	0.31 ± 0.007^g–k^	1.30 ± 0.029^l^	12.40 ± 0.279^ab^	0.25 ± 0.010^n^	71.71 ± 1.613^ab^
FB‐1	12.65 ± 0.225^cd^	0.29 ± 0.005^n^	1.51 ± 0.019^h^	12.12 ± 0.187^b–d^	1.94 ± 0.040^d^	72.96 ± 1.064^a^
FB‐2	12.690 ± 0.198^cd^	0.30 ± 0.005^i–m^	1.35 ± 0.022^k^	11.07 ± 0.173^g^	0.79 ± 0.053^l^	72.43 ± 1.138^a^
FB‐3	12.68 ± 0.334^cd^	0.33 ± 0.010^de^	1.36 ± 0.04^k^	11.81 ± 0.331^c–f^	0.69 ± 0.041^m^	66.79 ± 1.836^c^
FB‐4	13.52 ± 0.278^b^	0.45 ± 0.009^a^	2.53 ± 0.053^a^	12.61 ± 0.255^a^	3.19 ± 0.082^a^	67.07 ± 1.379^c^
FB‐5	12.31 ± 0.32^d^	0.33 ± 0.011^c–e^	1.59 ± 0.059^e–g^	11.13 ± 0.311^g^	1.55 ± 0.097^ij^	72.12 ± 2.259^ab^
FB‐6	12.73 ± 0.236^cd^	0.31 ± 0.006^g–j^	1.58 ± 0.024^g^	12.03 ± 0.206^b–e^	1.82 ± 0.040^ef^	71.70 ± 1.149^ab^
FB‐7	12.61 ± 0.241^cd^	0.29 ± 0.006^l–n^	1.49 ± 0.025^hi^	11.88 ± 0.211^c–f^	1.66 ± 0.037^g^	72.20 ± 1.237^a^
FB‐8	12.69 ± 0.250^cd^	0.31 ± 0.006^g–i^	1.61 ± 0.028^e–g^	12.04 ± 0.220^b–e^	1.89 ± 0.040^de^	71.67 ± 1.292^ab^
FB‐9	12.69 ± 0.247^cd^	0.31 ± 0.006^f–h^	1.59 ± 0.028^fg^	11.93 ± 0.218^c–f^	1.78 ± 0.041^f^	71.62 ± 1.268^ab^
SS	10.48	0.0707	3.9601	7.7144	24.513	162.000

*Note:* The superscript letters indicate statistically significant differences between the mean values within each column/row. Means that share the same superscript letter are not significantly different from each other, while means with different letters are significantly different at the chosen confidence level (e.g., *p* < 0.05).

### 3.4 Mineral Contents of Wheat Flour Blends Supplemented With Multi Cereals

The mineral compositions of wheat flour blends (FB‐0 to FB‐9) presented in (Table 5) demonstrated non‐significant differences when compared with standard wheat flour. Among macro minerals, Calcium level ranged from 870.42 to 926.50 ppm, while, potassium and sodium concentrations in the multi‐cereal flour varied between 763.08 to 848.33 ppm and 793.08 to 899.83 ppm respectively, highlighting mineral retention within different blends. Following a similar trend, micro minerals showed minimal differences ranging between (0.72 to 1.05 ppm; 1.63 to 2.19 ppm) for zinc and iron respectively. Similarly, heavy metals, like lead and nickel were observed within the safe limits ranging between (0.38 to 0.68 ppm) for lead, and (0.63 to 1.09 ppm) for nickel. Overall, all formulated blends showed statistically non‐significant variations comparable to standard wheat flour. This may be due to the removal of the pericarp layer of grains during milling which contains 75% of the minerals. However, all flour blends presented a slight increase in mineral profiles.

**TABLE 5:** Mineral composition (ppm) of wheat flour blends supplemented with multi‐cereal.

FB	Calcium (ppm)	Potassium (ppm)	Sodium (ppm)	Zinc (ppm)	Iron (ppm)	Lead (ppm)	Nickel (ppm)
FB‐0	977.66 ± 3.65	848.33 ± 7.52	875.33 ± 4.11	0.54 ± 0.03	1.63 ± 0.94	0.38 ± 0.20	0.84 ± 0.82
FB‐1	892.42 ± 3.62	830.50 ± 4.01	856.08 ± 9.98	0.78 ± 0.05	1.75 ± 0.83	0.52 ± 0.34	0.92 ± 0.84
FB‐2	870.42 ± 2.09	777.50 ± 8.14	830.33 ± 4.85	0.73 ± 0.03	2.19 ± 1.27	0.49 ± 0.30	1.09 ± 1.16
FB‐3	862.42 ± 9.78	826.33 ± 2.09	899.83 ± 4.33	0.91 ± 0.06	1.82 ± 0.79	0.60 ± 0.40	0.97 ± 0.85
FB‐4	896.17 ± 4.87	763.08 ± 4.12	875.00 ± 4.14	0.72 ± 0.04	1.63 ± 0.79	0.49 ± 0.29	0.86 ± 0.78
FB‐5	926.50 ± 8.71	800.50 ± 6.54	793.08 ± 2.77	1.05 ± 0.06	1.86 ± 0.70	0.68 ± 0.48	0.63 ± 0.62
FB‐6	887.59 ± 7.63	818.04 ± 4.32	859.77 ± 8.27	0.78 ± 0.05	1.78 ± 0.86	0.53 ± 0.34	0.94 ± 0.86
FB‐7	890.625 ± 8.12	821.78 ± 4.03	851.58 ± 8.04	0.82 ± 0.05	1.81 ± 0.86	0.54 ± 0.36	0.92 ± 0.85
FB‐8	893.20 ± 7.49	820.34 ± 4.13	856.05 ± 7.97	0.82 ± 0.05	1.75 ± 0.81	0.54 ± 0.36	0.89 ± 0.81
FB‐9	891.00 ± 6.14	815.04 ± 4.75	853.47 ± 2.46	0.81 ± 0.05	1.79 ± 0.86	0.54 ± 0.35	0.91 ± 0.84
SS	31301.0	19970	26700.5	0.51839	0.7882	0.17981	0.4424

### 3.5 Internal and External Characteristics of Wheat Flour Blend

Wheat flour supplemented with selected cereals was analyzed to assess parameters such as loaf volume, crust color, form symmetry, bake evenness, crust character, break and shred, and internal score, when compared with standard wheat flour. The findings presented in (Table 6) demonstrated noticeable variations among different treatments. FB‐0 showed the maximum loaf volume (8.90 ± 0.30 cm^3^/g), whereas the lowest loaf volume was noted in FB‐2 (6.10 ± 0.08 cm^3^/g). However, the standard sample (SS) showed the highest loaf volume when treatments were compared with the control. In terms of crust color, FB‐0 displayed the deepest hue (7.20 ± 0.31) and FB‐1 showed a softer hue (7.30 ± 0.31). On a similar pattern, form symmetry and bake evenness were seen highest in the FB‐0 blend (2.60 ± 0.14), when compared with other treatment blends. Similarly, crust character and internal score were observed highest in FB‐6 (3.10 ± 1.29; 45.70 ± 1.35) respectively. Overall, FB‐0 showed the highest sensory score across all parameters which confirms its suitability in baking quality; however, FB‐2 consistently demonstrated lower sensory results overall, indicating its weaker sensory properties. On the other hand, FB‐6 exhibited exceptional crust and internal quality with improved symmetry and uniform baking.

**TABLE 6:** Internal and external characteristics of wheat flour blends.

Cereals	Loaf volume (cm^3^/g)	Color of crust	Symmetry of form	Evenness of bake	Crust character	Break and shred	Intern score
FB‐0	8.90 ± 0.30^a^	7.20 ± 0.31^a^	2.60 ± 0.14^a^	2.50 ± 0.08^a^	3.30 ± 0.07^bc^	2.20 ± 0.08^de^	26.70 ± 0.82^b^
FB‐1	8.90 ± 0.22^b^	7.30 ± 0.31^de^	3.10 ± 0.04^ef^	3.30 ± 0.09^b^	3.20 ± 0.09^b–d^	3.40 ± 0.09^cd^	23.70 ± 0.54^cd^
FB‐2	6.10 ± 0.08^l^	6.30 ± 0.04^ij^	3.10 ± 0.09^ef^	2.90 ± 0.06^de^	3.20 ± 0.13^b–d^	3.10 ± 0.01^fg^	19.30 ± 0.14^m^
FB‐3	7.30 ± 0.36^h–k^	6.60 ± 0.24^gi^	3.40 ± 0.04^bc^	3.30 ± 0.06^b^	2.90 ± 0.05^de^	2.90 ± 0.08^hi^	20.90 ± 0.26^j–l^
FB‐4	7.60 ± 0.05^f–h^	6.50 ± 0.08^hj^	3.20 ± 0.11^de^	2.90 ± 0.13^cd^	2.60 ± 0.03^f^	3.10 ± 0.08^fg^	20.50 ± 0.19^l^
FB‐5	7.80 ± 0.04^e–g^	6.30 ± 0.12^ij^	3.20 ± 0.12^de^	3.20 ± 0.12^bc^	2.80 ± 0.13^ef^	3.30 ± 0.11^de^	21.10 ± 0.36^i–l^
FB‐6	7.50 ± 0.18^g–i^	7.60 ± 0.33^cd^	3.10 ± 0.08^ef^	3.20 ± 0.07^bc^	3.10 ± 1.29^a^	3.70 ± 0.15^a^	45.70 ± 1.35^a^
FB‐7	7.20 ± 0.19^h–k^	7.30 ± 0.43^de^	3.10 ± 0.06^ef^	3.60 ± 0.04^a^	3.20 ± 0.07^b–d^	2.90 ± 0.12^gh^	21.90 ± 0.66^f–h^
FB‐8	7.40 ± 0.24^g–j^	8.20 ± 0.29^a^	3.20 ± 0.11^de^	3.30 ± 0.04^b^	3.40 ± 0.13^bc^	3.60 ± 0.05^ab^	23.60 ± 0.54^cd^
FB‐9	7.80 ± 0.43^e–g^	7.90 ± 0.09^ab^	3.10 ± 0.04^ef^	3.50 ± 0.12^a^	3.50 ± 0.12^b^	3.20 ± 0.12^ef^	23.60 ± 0.37^cd^
SS	72.495	48.0288	3.106	4.296	2530.515	9.934	3004.824

*Note:* The superscript letters indicate statistically significant differences between the mean values within each column/row. Means that share the same superscript letter are not significantly different from each other, while means with different letters are significantly different at the chosen confidence level (e.g., *p* < 0.05).

The reduced specific volume of bread dough may be attributed to a reduced gluten network and impaired gas retention. The color changes may be due to the Maillard reaction in the presence of wheat flour substitutes greater than 10% (Wang et al. 2018). Similar results were presented by Al‐Attabi et al. (2017), who stated that barley flour supplementation in wheat flour caused the reduction of gluten and the overall texture of bread dough. On the other hand, the findings of Koletta et al. (2014), who investigated the nutritional quality of composite flour containing barley, oat and rye flour showed richer contents of dietary fiber which resulted in increased hardness and chewiness; whereas, up to 60% substitution of wheat flour with multigrain presented a limited gluten matrix that reduced the elasticity and resilience of dough.

### 3.6 Evaluation of Sensory Parameters of Wheat Flour Blends

Organoleptic properties of bread dough play a vital role in consumer preference which depends on the grains used for bread making. The sensory score of wheat flour supplemented with multi‐cereal blends was evaluated on a nine‐point hedonic scale, which was assessed by departmental trained panel members. Incorporation of different cereals and grains in whole wheat flour imparts unfavorable changes in bread properties including color, texture, chewability and flavor. However, research studies are done to optimize combinations of multi‐cereal incorporation in whole wheat flour.

Sensory attributes presented in (Table 7) indicated significant differences among all blends for all measurable attributes including grain, crumb, color, aroma, mastication, taste, texture and external score. FB‐0, containing 100% single grain flour consistently showed the highest sensory score with grain (8.60 ± 0.42), crumb color (9.10 ± 0.49), aroma (13.50 ± 0.84), mastication (14.50 ± 0.56), taste (13.00 ± 0.59), texture (8.80 ± 0.10), and external score (67.50 ± 1.06). The uniform structure and palatability in the FB‐0 blend suggest optimal gluten development responsible for maintaining loaf volume and crumb evenness. FB‐1 (75% SGF and 25% barley) and FB‐4 (75% SGF and 25% oat) showed satisfactory texture, crumb softness and overall acceptability. However, FB‐2 containing (75% SGF and 25% corn) showed a slight coarse texture due to reduced gluten development. In contrast, FB‐3, containing 25% millet flour showed consistently poor sensory values with grain (5.70 ± 0.29), crumb color (6.70 ± 0.08), aroma (5.10 ± 0.23), mastication (9.30 ± 0.33), taste (8.60 ± 0.32), texture (5.90 ± 0.10), external score (40.90 ± 0.58). The poor sensory attributes suggest that supplementation of millet flour, rich in high fiber adversely affects dough handling and crumb formation which in turn affects taste, texture and mouthfeel. Similarly, among multi‐cereal combinations, FB‐6 relatively balanced aroma, taste and texture with moderate proportions of barley, corn, millet and oat. However, FB‐7 FB‐8 and FB‐9 showed reduced color and texture scores due to the combined effect of multiple coarse cereals affecting loaf volume and crumb.

**TABLE 7:** Sensory evaluation of the wheat flour bread.

Cereals	Grain	Color of crumb	Aroma	Mastication	Taste	Texture	Extern score
FB‐0	8.60 ± 0.42^a^	9.10 ± 0.49^a^	13.50 ± 0.84^a^	14.50 ± 0.56^a^	13.00 ± 0.59^a^	8.80 ± 0.10^a^	67.50 ± 1.06^a^
FB‐1	8.20 ± 0.39^b^	7.60 ± 0.26^b^	12.60 ± 0.39^b^	12.76 ± 0.33^b^	11.90 ± 0.35^b^	7.40 ± 0.24^b^	59.90 ± 0.69^b^
FB‐2	6.50 ± 0.32^ef^	7.60 ± 0.32^b^	11.60 ± 0.68^c^	10.10 ± 0.25^c^	9.60 ± 0.55^f^	6.30 ± 0.15^g–i^	51.80 ± 0.99^d^
FB‐3	5.70 ± 0.29^g^	6.70 ± 0.08^c–g^	5.10 ± 0.23^k^	9.30 ± 0.33^e–g^	8.70 ± 0.32^g^	5.90 ± 0.11^i–k^	40.90 ± 0.58^m^
FB‐4	6.30 ± 0.07^ef^	6.60 ± 0.34^d–h^	6.20 ± 0.33^j^	9.20 ± 0.21^fg^	9.60 ± 0.59^f^	5.80 ± 0.25^j–l^	43.20 ± 0.57^k^
FB‐5	6.50 ± 0.15^ef^	6.30 ± 0.32^g–k^	9.60 ± 0.26^e^	8.20 ± 0.18^ij^	10.60 ± 0.41^d^	6.30 ± 0.33^g–i^	46.90 ± 0.36^h^
FB‐6	6.60 ± 0.05^d–f^	7.10 ± 0.14^c^	7.60 ± 0.38^i^	7.90 ± 0.41^jk^	8.50 ± 0.12^gh^	6.20 ± 0.35^hi^	43.40 ± 0.63^k^
FB‐7	6.60 ± 0.22^d–f^	6.50 ± 0.08^e–i^	8.10 ± 0.34^hi^	8.30 ± 0.09^ij^	8.30 ± 0.48^g–i^	5.90 ± 0.13^i–k^	43.30 ± 0.25^k^
FB‐8	6.50 ± 0.25^ef^	6.30 ± 0.23^g–k^	8.60 ± 0.39^gh^	7.90 ± 0.29^jk^	8.20 ± 0.28^g–i^	5.60 ± 0.18^l^	42.70 ± 0.35^kl^
FB‐9	6.50 ± 0.11^ef^	6.10 ± 0.17^i–k^	8.10 ± 0.39^hi^	7.60 ± 0.19^k^	7.90 ± 0.19^h–j^	6.10 ± 0.35^ij^	41.9 ± 0.97^lm^
SS	65.428	66.642	426.020	313.844	282.495	89.541	4499.790

*Note:* The superscript letters indicate statistically significant differences between the mean values within each column/row. Means that share the same superscript letter are not significantly different from each other, while means with different letters are significantly different at the chosen confidence level (e.g., *p* < 0.05).

Wheat flour substitution presents an off‐flavor which is responsible for a major limitation in consumer preference and acceptability. Therefore, optimization of specific ingredients is required to obtain the desired taste and overall acceptability. Aly et al. (2021), stated that 40% whole barley flour (WBF) supplemented in wheat biscuits showed a decline in color and surface appearance of biscuits. Due to the presence of β‐Glucan, barley‐based bread offers reduced loaf volume, crumb and taste (del Carmen Robles‐Ramírez et al. 2020). The results were endorsed by findings of Wang et al. (2018), who presented that the incorporation of 5%–10% isolated barley protein in wheat flour bread induced an intense bitter taste. Similarly, the addition of 50% sorghum flour to wheat flour confers a bitter taste (Kotsiou et al. 2022).

### 3.7 Dough Characteristics of Wheat Flour Dough

Sensory characteristics of wheat flour supplemented with multi‐cereal blends presented in (Table 8) revealed considerable variations in sensory and rheological characteristics including smoothness, extensibility/elasticity, stickiness, color, dryness and stiffness among different blends. Wheat flour substitutes generally led to a decrease in the functional properties and rheological behavior of bread. Generally, more than 10% substitution of wheat flour substitutes offers challenges, that is, longer dough development time, reduced dough extensibility, starch gelatinization, decreased gluten strength and stickiness.

**TABLE 8:** Dough characteristics of wheat flour dough characters.

Cereals	Smoothness	Elasticity	Stickiness	Color	Dryness	Stiffness
FB‐0	8.80 ± 0.44^a^	8.60 ± 0.27^a^	9.30 ± 0.64^a^	8.80 ± 0.31^a^	8.90 ± 0.33^a^	8.10 ± 0.37^a^
FB‐1	5.90 ± 0.17^g–i^	5.00 ± 0.21^lm^	6.95 ± 0.45^de^	7.00 ± 0.21^c–e^	7.70 ± 0.37^b^	7.30 ± 0.21^bc^
FB‐2	7.40 ± 0.22^c^	5.80 ± 0.24^ij^	5.30 ± 0.08^j^	6.00 ± 0.29^i^	4.80 ± 0.12^hi^	6.430 ± 0.07^ef^
FB‐3	6.30 ± 0.20^e–g^	5.30 ± 0.23^k^	7.40 ± 0.28^c^	6.50 ± 0.32^f–h^	7.40 ± 0.47^bc^	5.40 ± 0.05^i^
FB‐4	6.90 ± 0.46^d^	6.20 ± 0.40^h^	6.40 ± 0.12^fg^	7.20 ± 0.25^b–d^	6.80 ± 0.20^d^	6.70 ± 0.13^de^
FB‐5	6.00 ± 0.47^g–i^	5.00 ± 0.24^k–m^	6.90 ± 0.09^de^	6.20 ± 0.18^hi^	6.00 ± 0.10^ef^	7.50 ± 0.41^b^
FB‐6	7.40 ± 0.44^c^	6.10 ± 0.44^hi^	8.40 ± 0.18^b^	4.80 ± 0.06^k^	5.90 ± 0.24^ef^	5.40 ± 0.09^i^
FB‐7	6.60 ± 0.48^d–f^	5.20 ± 0.18^kl^	6.20 ± 0.28^g^	6.30 ± 0.37^g–i^	7.30 ± 0.12^bc^	5.30 ± 0.21^i^
FB‐8	7.00 ± 0.15^cd^	7.60 ± 0.27^b^	8.50 ± 0.12^b^	7.00 ± 0.14^c–e^	5.10 ± 0.11^gh^	5.90 ± 0.29^gh^
FB‐9	8.20 ± 0.48^b^	6.30 ± 0.34^gh^	6.20 ± 0.32^g^	6.10 ± 0.11^hi^	7.00 ± 0.28^cd^	4.10 ± 0.26^k^
SS	197.174	147.170	217.888	268.195	249.787	193.037

*Note:* The superscript letters indicate statistically significant differences between the mean values within each column/row. Means that share the same superscript letter are not significantly different from each other, while means with different letters are significantly different at the chosen confidence level (e.g., *p* < 0.05).

FB‐0 showed maximum sensory attributes in terms of smoothness (8.80 ± 0.44) mainly due to a strong gluten network in 100% wheat flour. Moreover, maximum elasticity and stickiness were observed in the FB‐0 blend. FB‐1 and FB‐3 supplemented with barley and millet respectively showed reduced smoothness and elasticity values (5.90 ± 0.17; 5.30 ± 0.23), reflecting limited gluten development due to high fiber content. The FB‐1 blend of wheat flour supplemented with 25% barley showed improved bread volume, aroma and overall sensory characteristics. Similarly, FB‐2, and FB‐3 containing corn and millet showed reduced color due to the presence of pigment compounds including carotenoids. In addition to this, reduced elasticity and stickiness values were observed in these blends due to the presence of non‐gluten proteins. The addition of oats and sorghum in FB‐4 and FB‐5 showed improved smoothness and texture due to the presence of soluble fiber. Among composite flours, FB‐9 (multigrain mix) showed moderate stiffness and smoothness in dough. Likewise, FB‐8 showed higher dryness. Overall, all composite blends demonstrated reduced stiffness and elasticity without altering dough functionality.

Reduced elasticity and mixing tolerance was observed in FB‐2 and FB‐3 suggesting weakening of dough which may possess mechanical resistance during mixing, kneading and dough development. The findings were found compatible with the results of Olagunju et al. 2021, who supplemented wheat flour with amaranth and acha flour. Similarly, 15%–20% barley substitution can improve crumb softness, mouth feel and viscosity due to the presence of β‐glucans (El‐Taib et al. 2018). However, lack of gluten in corn reduced the elasticity and cohesiveness of bread dough. The findings were supported by Fu et al. 2016, who documented that composite dough having extruded corn flour showed a downward trend in dough development time and dough stability, due to a broken gluten matrix and weak thermal viscosity. Another study conducted by Raya et al. (2022) showed a decline in dough stability and extensibility with a gradual increase in the level of corn flour substitution in the wheat flour blend. Similarly, the blend supplemented with millet (FB‐3) showed a lower elasticity score which may be due to a reduced gluten network and its fiber. However, dull crust color may be due to the presence of pigments responsible for darker color and bitter taste (Ovsiannykova et al. 2019). Another study presented decreased volume and crust in composite flour supplemented with millet with a gradual increase in millet flour (Man et al. 2016). Similarly, Dubey et al. 2025, reported that the least chewiness value was found in the composite flour blend prepared by the incorporation of sorghum and millet flour in equal proportion, thus affecting the textural profile of the flour blend. The sensorial attributes including dough color were affected through a gradual increase in sorghum flour substitution in wheat flour (Sibanda et al. 2015). Moreover, the resilience of the bread was greatly reduced by oat flour, due to an altered gluten matrix as reported by Tiwari et al. (2013). Malik et al. (2016) stated that the incorporation of oat, barley, maize, rice and flax seeds in wheat flour enhanced the firmness and chewiness of composite flour; however with an increase in their percentage in wheat, it reduced the specific volume of dough. Similarly, more than 15% addition of soybean and chickpea flour in wheat flour reduces the viscoelasticity of dough. Incorporation of amaranth flour into whole wheat flour affects dough development stability due to the weakening of the gluten matrix (Mir et al. 2023).

## 4 Conclusion

The blending of barley, sorghum, millet and oat flours with the wheat flour significantly enhanced the nutritional profile of composite breads by increasing protein, mineral and antioxidant activity while maintaining the acceptability of sensory attributes and dough rheology with up to 20% substitution. These results provide new insights for the multi‐grain optimization of blends for functional and consumer‐friendly products. However, this work was limited to the trials without determining the glycemic response, and storage stability. Future research should be focused on exploring protein‐starch interaction and bioavailability assessment. Overall, multi‐cereal integration represents a health‐oriented and sustainable approach to the development of nutritionally rich composite products aligned with current sustainable and dietary goals.

## Author Contributions

**Farooq Hafeez:** formal analysis (equal), writing – original draft (equal), methodology (equal). **Muhammad Tauseef Sultan:** conceptualization (equal), supervision (lead), data curation (equal). **Hassan Raza:** writing – original draft (equal), editing – original draft (equal). **Muhammad Usman Khalid:** editing – original draft (equal). **Ali Ikram:** editing – original draft (equal). **Muhammad Tayyab Arshad:** validation (equal). **Iqra Baig:** writing – original draft (equal), visualization (equal). **Sidra Imtiaz:** editing – original draft (equal). **Muhammad Abrar:** formal analysis (equal). **Ali Imran:** visualization (equal), editing – original draft (equal). **Kodjo Théodore Gnedeka:** visualization (equal).

## Funding

The authors have nothing to report.

## Ethics Statement

The authors have nothing to report.

## Consent

The authors have nothing to report.

## Conflicts of Interest

The authors declare no conflicts of interest.

## Data Availability

The data that support the findings of this study are available from the corresponding authors upon reasonable request.
